# West Midlands general adult psychiatry higher trainees’ peer group wellbeing away day

**DOI:** 10.1192/bjo.2021.387

**Published:** 2021-06-18

**Authors:** Emma Fisher

**Affiliations:** Coventry and Warwickshire Partnership Trust

## Abstract

**Aims:**

The main aim of the wellbeing day was to increase the sense of wellbeing amongst psychiatry higher trainees in the West Midlands. We first wanted to understand the wellbeing needs of the trainees and what they hoped to get out of an away day. We wanted then to evaluate whether the away day had met these needs.

**Background:**

The Psychiatry Trainees Committee (PTC) published a report ‘Supported and Valued? A trainee led review into morale and training within psychiatry’ in 2017. The importance of feeling valued and supported and the value trainees place upon the support of their peers, were highlighted in this report.

As higher trainees we are often geographically isolated from each other, and whilst the peer group meet once per month, this is mostly for academic lectures resulting in poor familiarity amongst trainees which can leave trainees feeling unknown and unsupported.

**Method:**

We decided to apply to HEE for funding for an away day. We surveyed the peer group, asking what they most wanted to get out of an away day. The results showed that ‘a morale boost’, ‘destress/relaxation’ and ‘opportunity to get to know other trainees’ were the trainee's priorities, followed by improving leadership, team working and negotiation skills.

With these priorities in mind, an away day programme was developed which included a talk from Dr Mike Blaber, a palliative care doctor with a special interest in doctors’ wellbeing, a ‘getting to know you’ art activity and a team building GPS treasure hunt funded by HEE. The day finished with a dinner in a local restaurant sponsored by Recordati. The rest of the day was paid for by the peer group.

**Result:**

28 higher trainees attended the away day which was held in Birmingham on 11/07/2019. Trainees gave feedback on the day using an online anonymous survey. 81% of attendees said the away day decreased their stress levels. 90% said that the day had increased their sense of wellbeing. 86% felt an increased sense of belonging and less isolated as a trainee.

**Conclusion:**

Regular trainee away days may help tackle isolation and increase morale which is linked to better patient outcomes. Improving trainees’ sense of wellbeing leads to better job satisfaction, which may ultimately lead to higher rates of retention within psychiatry.

